# Sustainable Coating Based on Zwitterionic Functionalized Polyurushiol with Antifouling and Antibacterial Properties

**DOI:** 10.3390/molecules28248040

**Published:** 2023-12-11

**Authors:** Kaiyue Xu, Huimin Xie, Chenyi Sun, Wenyan Lin, Zixuan You, Guocai Zheng, Xiaoxiao Zheng, Yanlian Xu, Jipeng Chen, Fengcai Lin

**Affiliations:** Fujian Engineering and Research Center of New Chinese Lacquer Materials, College of Materials and Chemical Engineering, Minjiang University, Fuzhou 350108, China; xukaiyue@stu.mju.edu.cn (K.X.); xiehuimin@stu.mju.edu.cn (H.X.); sunchenyi@stu.mju.edu.cn (C.S.); linwenyan@stu.mju.edu.cn (W.L.); youzixuan@stu.mju.edu.cn (Z.Y.); 2231@mju.edu.cn (G.Z.); xxzheng@mju.edu.cn (X.Z.); ylxu@mju.edu.cn (Y.X.)

**Keywords:** urushiol, zwitterionic polymers, marine antifouling coating, biomass-based coating

## Abstract

Zwitterionic polymer coatings facilitate the formation of hydration layers via electrostatic interactions on their surfaces and have demonstrated efficacy in preventing biofouling. They have emerged as a promising class of marine antifouling materials. However, designing multifunctional, environmentally friendly, and natural products-derived zwitterionic polymer coatings that simultaneously resist biofouling, inhibit protein adhesion, exhibit strong antibacterial properties, and reduce algal adhesion is a significant challenge. This study employed two diisocyanates as crosslinkers and natural urushiol and ethanolamine as raw materials. The coupling reaction of diisocyanates with hydroxyl groups was employed to synthesize urushiol-based precursors. Subsequently, sulfobetaine moieties were introduced into the urushiol-based precursors, developing two environmentally friendly and high-performance zwitterionic-functionalized polyurushiol antifouling coatings, denoted as HUDM-SB and IPUDM-SB. The sulfobetaine-functionalized polyurushiol coating exhibited significantly enhanced hydrophilicity, with the static water contact angle reduced to less than 60°, and demonstrated excellent resistance to protein adhesion. IPUDM-SB exhibited antibacterial efficacy up to 99.9% against common Gram-negative bacteria (*E. coli* and *V. alginolyticus*) and Gram-positive bacteria (*S. aureus* and *Bacillus*. sp.). HUDM-SB achieved antibacterial efficacy exceeding 95.0% against four bacterial species. Furthermore, the sulfobetaine moieties on the surfaces of the IPUDM-SB and HUDM-SB coatings effectively inhibited the growth and reproduction of algal cells by preventing microalgae adhesion. This zwitterionic-functionalized polyurushiol coating does not contain antifouling agents, making it a green, environmentally friendly, and high-performance biomaterial-based solution for marine antifouling.

## 1. Introduction

The attachment and growth of microorganisms, algae, barnacles, and other aquatic organisms on the surfaces of ships, marine facilities, or other underwater structures in the marine environment is referred to as marine biofouling [1,2]. A globally recognized issue, biofouling leads to surface contamination of vessels and underwater equipment, increased hydrodynamic resistance, corrosion of underwater structures, threats to safe operations, economic losses, and environmental concerns [3]. These challenges significantly impede the development and utilization of marine resources worldwide. Protective coatings are the most direct and effective means of mitigating marine biofouling. However, the marine antifouling coatings used domestically and internationally often rely on petroleum-based resin materials containing toxic metal ions and bioactive additives, such as Cu_2_O [4,5], zinc pyrithione [6,7], and copper/zinc pyrithione sulfate [8]. Although they combat marine biofouling, the self-degradation products of these coatings can severely harm non-target organisms and the marine environment. Consequently, within the framework of achieving global “carbon neutrality”, there is a pressing need to design and synthesize environmentally friendly, non-toxic, renewable, and high-performance marine antifouling coatings based on natural extracts (such as cellulose [9,10], urushiol [11], tannic acid [12], and tung oil [13]).

Hydrophilic materials that are resistant to protein adhesion represent a prominent category of fouling-resistant coatings. This property is often achieved through the incorporation of polyethylene glycol (PEG) and zwitterions to enhance the hydrophilicity of the material [14,15]. PEG is a non-toxic material that forms hydration layers through extensive hydrogen bonding with water molecules and effectively inhibits protein adhesion [14,16]. However, PEG lacks inherent biocidal properties and has insufficient mechanical performance to support its use as a long-term antifouling coating. Zwitterionic compounds are an alternative class of hydrophilic antifouling material featuring chemical structures that contain positively and negatively charged groups [17]. These compounds undergo electrostatic interactions to form hydration layers and display strong hydrophilicity, deterring the adhesion of fouling organisms via inhibition of protein adhesion [18].Typical cationic zwitterions include quaternary ammonium ions, while anionic zwitterions include sulfobetaines, carboxybetaines, and phosphocholines [18,19]. Zwitterions can be utilized to modify surface grafting to tailor the surface properties of polymers and achieve high hydrophilicity in their coatings. Additionally, the quaternary ammonium ions in zwitterionic compounds impart effective antibacterial properties [20,21]. Therefore, zwitterion-modified polymer surfaces form hydration layers on the coating surfaces to prevent adhesion by fouling organisms and also exhibit biocidal effects due to the quaternary ammonium groups. The synergistic action of these two mechanisms gives these coatings exceptional antifouling characteristics. Zhang et al. [22] employed a one-pot reaction to graft zwitterionic esters and capsaicin polymer on a polydimethylsiloxane (PDMS) network, resulting in a multifunctional marine antifouling coating with excellent adhesion resistance for protein, bacteria, and diatoms. Guan et al. [23] significantly enhanced the surface hydrophilicity of modified membranes using layer-by-layer interfacial polymerization of zwitterionic polymers, concurrently increasing water permeability to create a material with outstanding antifouling properties. The coatings do not release other antifouling agents because the quaternary ammonium groups are grafted onto them. Therefore, zwitterion surface functionalization offers an environmentally friendly and synergistic antifouling strategy. Although the exceptional performance of zwitterionic compounds in marine antifouling has been confirmed, their independent application in marine antifouling is challenging. Firm coupling with substrate materials through chemical or physical methods is required to fully leverage their excellent antibacterial and antifouling properties.

Raw lacquer, a distinctive and high-quality natural resource in Asia, is a natural resin coating with excellent adhesion, film-forming properties, renewability, and environmental friendliness [24,25]. It has been used as an adhesive and coating for furniture, wooden structures, and other items for thousands of years. Urushiol is the primary component of raw lacquer and has a catechol-like ortho-quinone structure with alkyl side chains of C_15_–C_17_ on the benzene ring [26]. Urushiol is a premium natural resource for constructing green marine antifouling coatings because the dual-active hydroxyl and unsaturated double bonds within its molecular structure render it highly chemically reactive and modifiable [11]. The active phenolic hydroxyl group of urushiol can readily form a stable interface with the functional groups of zwitterionic compounds through covalent or non-covalent bonds. This interaction creates active sites with high potential for adhesion in zwitterionic compounds. The flexible, unsaturated long side-chain structure of urushiol can undergo a self-polymerization reaction, leading to the formation of a mesh polymer cross-linking network with favorable mechanical properties for use in zwitterionic polymer coatings. Urushiol polymer coatings have a smooth and compact texture, high hardness, excellent stability, and resistance to organic solvents and chemical corrosion. These properties may collectively enhance the underwater stability of zwitterionic polymer coatings. Furthermore, the structure and properties of urushiol-based polymer coatings can be effectively adjusted by altering the ratio of the components. This flexibility enables the creation of a novel, green, bio-based marine antifouling coating with superior performance.

In the present study, environmentally friendly sulfobetaine-functionalized polyurushiol coatings, specifically HUDM-SB and IPUDM-SB, were synthesized using natural urushiol and ethanolamine as raw materials. Two types of diisocyanates were selected as cross-linking agents, and the coupling reaction of diisocyanates with the hydroxyl groups of urushiol was employed to synthesize urushiol-based precursors. Subsequently, sulfobetaine groups were introduced into these precursors, resulting in two environmentally friendly and high-performance zwitterionic-functionalized urushiol-polymer antifouling coatings. The impact of zwitterionic groups on the wettability, thermal stability, and physical–mechanical properties of the coatings was investigated using contact angle measurements, liquid-droplet adhesion tests, thermogravimetric analysis, and tests of mechanical properties. Furthermore, bovine serum albumin (BSA) and γ-globulin were used to systematically evaluate the ability of these coatings to resist protein adhesion. Additionally, the antibacterial and anti-diatom-fouling performances of the coatings were comprehensively examined using the Gram-negative bacteria *Escherichia coli* (*E. coli*) and *Vibrio alginolyticus* (*V. alginolyticus*), the Gram-positive bacteria *Staphylococcus aureus* (*S. aureus*) and *Bacillus species* (*Bacillus*. Sp.), and the algae *Nitzschia closterium* (*N. closterium*) and *Phaeodactylum tricornutum* (*P. tricornutum*). These coatings were primarily sourced from urushiol, a natural product, and sulfobetaine zwitterionic groups and contained no additional fouling agents. The coatings offer a novel approach to developing green, environmentally friendly, and high-performance biomaterial-based marine antifouling materials.

## 2. Results and Discussions

### 2.1. Synthesis and Structural Characterization of the HUDM-SB and IPUDM-SB Monomers

**Figure 1:**
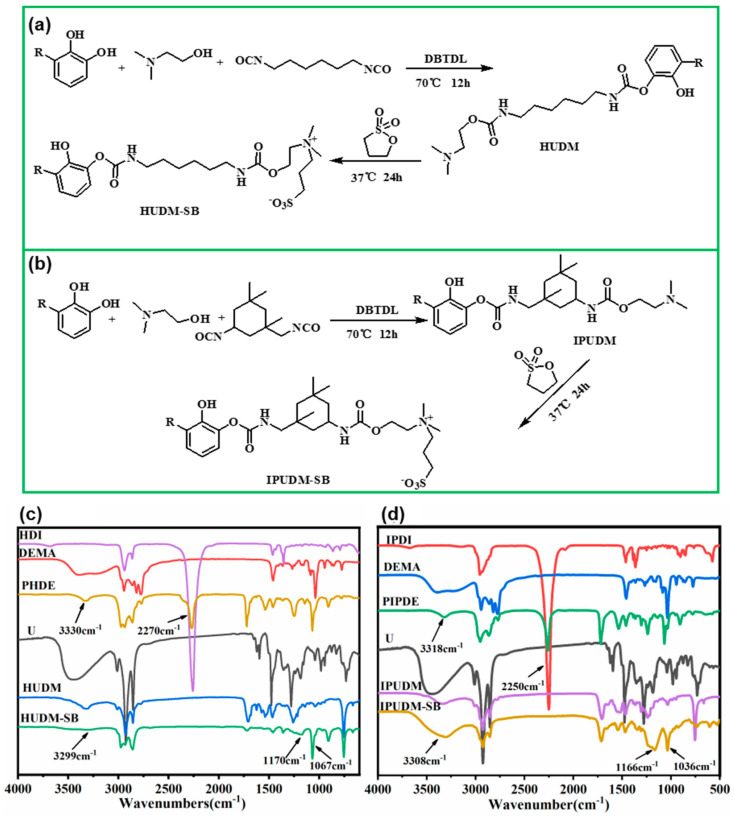
(**a**,**b**) Synthesis of HUDM, HUDM−SB, IPUDM and IPUDM−SB. ATR−FTIR spectra of (**c**) HUDM−SB and (**d**) IPUDM−SB.

As shown in Figure 1a, the reaction between DMEA and HMDI formed PHDE containing aliphatic isocyanate (NCO) groups. Subsequently, the NCO groups in PHDE reacted with OH groups in urushiol to produce a urushiol monomer with quaternary amine structures (HUDM). Next, zwitterionic sulfobetaine glycine monomers were covalently linked to HUDM molecules through an opening reaction with 1,3-propane sulfone and the tertiary amine, yielding HUDM-SB. The chemical structure of HUDM-SB was characterized using FTIR spectroscopy, as shown in Figure 1c. The FTIR spectrum of HMDI exhibited characteristic peaks near 2270 cm^−1^ corresponding to the NCO groups [27]. Additionally, NCO peaks were observed around 2270 cm^−1^ in the PHDE spectrum, albeit with reduced intensity compared to HMDI, indicating a reaction between a few NCO groups in HMDI and DMEA. However, the characteristic NCO peaks disappeared in the FTIR spectrum of HUDM, suggesting the reaction of the remaining isocyanate groups in HMDI with urushiol [28]. Furthermore, a series of characteristic absorption peaks corresponding to the phenolic amide ester linkages were observed, confirming the successful coupling of NCO and OH groups. In the PHDE and HUDM spectra, peaks at 3330 and 3299 cm^−1^ were attributed to the N-H stretching vibrations in amide esters and phenolic amide ester bonds [29], respectively. The peaks at 1721 and 1706 cm^−1^ corresponded to the C=O stretching vibrations [30], and overlapping peaks at 1249–1259 cm^−1^ indicated the stretching and bending vibrations of the C-N bond in the amide and N-H bonds [31]. Therefore, these results verified the successful synthesis of HUDM, the precursor containing aminoethyl methacrylate and phenolic amide ester bonds. Moreover, the FTIR spectrum of HUDM-SB displayed strong characteristic absorption peaks at 1170 and 1067 cm^−1^, corresponding to the vibrational absorption peaks of the –SO_3_^−^ groups, confirming the successful coupling of sulfobetaine glycine groups to the urushiol molecules [32].

Similarly, the reaction between DMEA and IPDI formed PIPDE containing NCO groups, as shown in Figure 1b. The NCO groups in PIPDE readily coupled with OH groups in urushiol molecules to yield IPUDM, which is urushiol with tertiary amine structures. Subsequently, zwitterionic sulfobetaine glycine monomers were covalently linked to urushiol through an opening reaction with 1,3-propane sulfone and the tertiary amine to form IPUDM-SB. The chemical structure of IPUDM-SB was characterized using FTIR spectroscopy, as shown in Figure 1d. Compared to the FTIR spectrum of IPDI shown in Figure 1d, the absorption peak intensity of the NCO characteristic peak around 2250 cm^−1^ in PIPDE was significantly reduced, indicating a reaction between the NCO groups in IPDI and DMEA [33]. The NCO characteristic peaks disappeared in the FTIR spectrum of IPUDM, suggesting a complete reaction of the two isocyanates in IPDI with DMEA and urushiol. The peaks at 3308 and 3318 cm^−1^ in the PIPDE and IPUDM spectra corresponded to the N-H stretching vibrations in amide ester and phenolic amide ester bonds, respectively. The peaks at 1719 and 1713 cm^−1^ represented the C=O stretching vibrations [34]. The overlapping peaks at 1237–1238 cm^−1^ indicated the stretching and bending vibrations of the C-N bond in the amide and N-H bonds. Therefore, these results confirmed the successful synthesis of IPUDM, the precursor containing aminoethyl methacrylate and phenolic amide ester bonds. Furthermore, the FTIR spectrum of IPUDM-SB showed strong characteristic absorption peaks at 1166 and 1036 cm^−1^, corresponding to the vibrational absorption peaks of the –SO_3_^−^ groups, confirming the successful coupling of sulfobetaine glycine groups with the urushiol molecules [32].

### 2.2. Surface Elemental Composition of the HUDM-SB and IPUDM-SB Coatings

**Figure 2:**
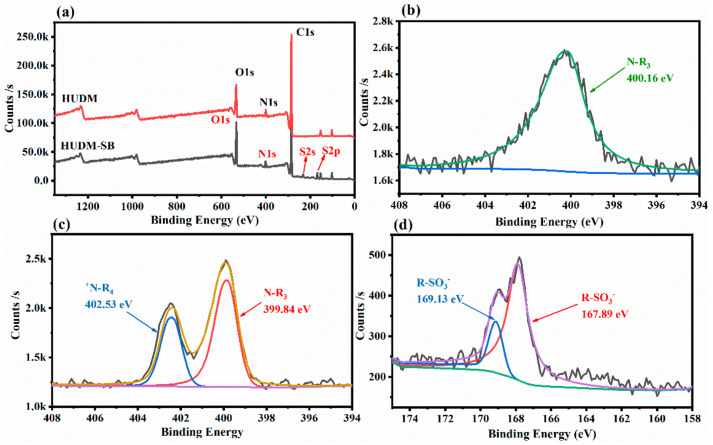

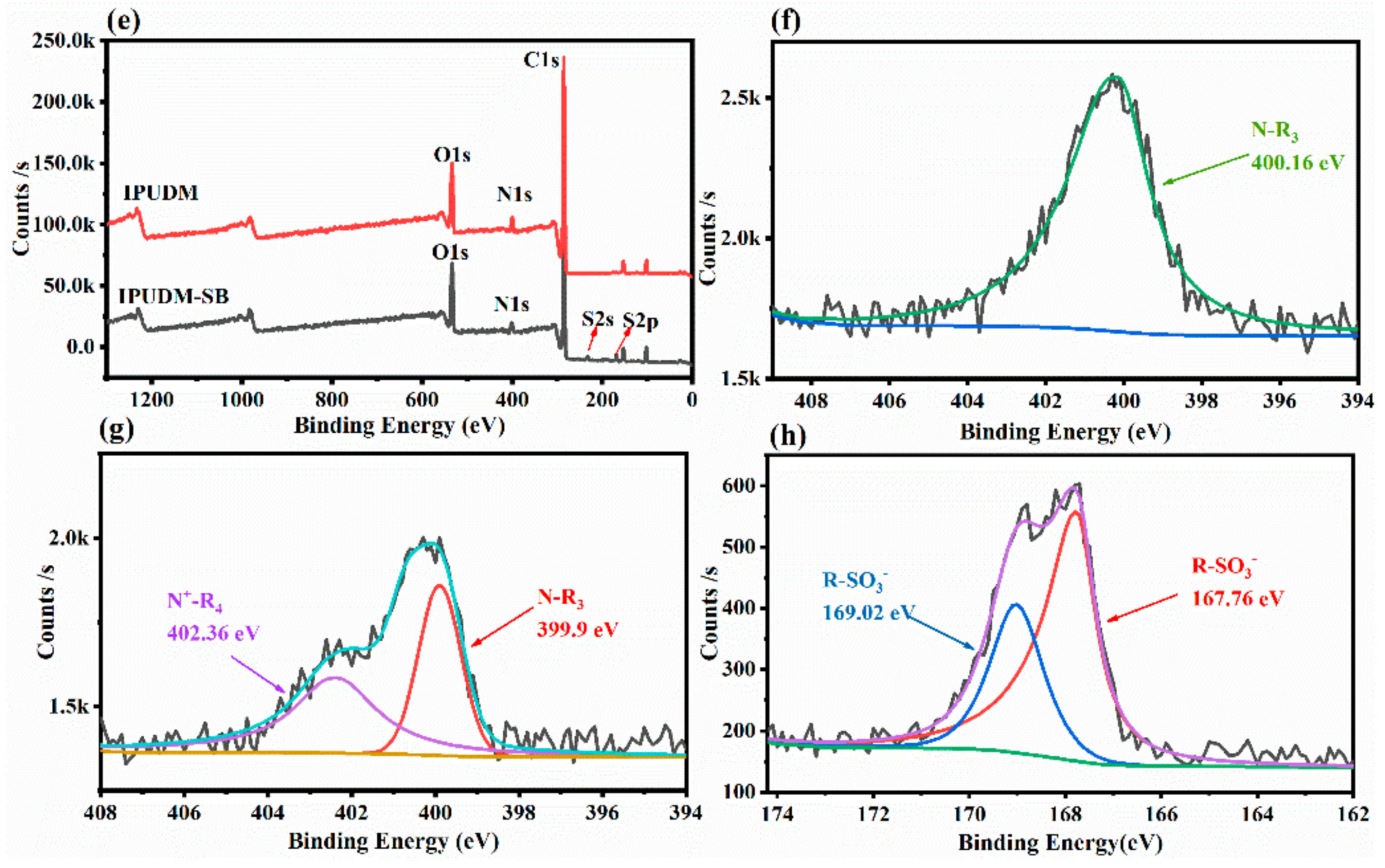
(**a**,**e**) XPS survey spectra stack graph of IPUDM, HUDM, IPUDM−SB and HUDM−SB. N1s core-grade XPS spectra of (**b**) HUDM, (**c**) HUDM−SB, (**f**) IPUDM, and (**g**) IPUDM−SB. S2p core-grade XPS spectra of (**d**) HUDM−SB and (**h**) IPUDM−SB.

As illustrated in Figure 2, XPS was employed to characterize the surface elemental composition of the zwitterion-functionalized urushiol polymer coatings. The XPS spectra of the IPUDM and HUDM coatings showed only three peaks, representing C1s (284.5 eV), N1s (400.1 eV), and O1s (544.9 eV). However, as shown in Figure 2a,e, additional characteristic peaks for S elements, S2p and S2s, were evident in the XPS spectra of IPUDM-SB and HUDM-SB coatings where sulfobetaine moieties were introduced, indicating the successful grafting of sulfobetaine to urushiol molecules [35]. As shown in the high-resolution N1s spectra in Figure 2b,f, IPUDM and HUDM coatings had a single primary peak at ~400.1 eV. As shown in Figure 2c,g, the N1s spectra of the IPUDM-SB coatings had two distinct peaks at 402.3 and 399.9 eV, and the N1s spectra of the HUDM-SB coatings had two distinct peaks at 402.5 and 399.8 eV. The peaks at 399.9 and 399.8 eV corresponded to the unreacted tertiary amines on the IPUDM-SB and HUDM-SB coatings, respectively. The other peaks at 402.5 and 402.3 eV represented the quaternary ammonium ions (+NR_4_) formed after the successful chemical grafting of 1,3-propane sulfone [32]. The quaternary ammonium ions required higher energy to displace electrons from their N1s orbitals due to their electron-deficient and more stable nature. Additionally, as shown in Figure 2d,h, the surfaces of the IPUDM-SB coatings had two sulfur (S) elemental peaks at 169.0 and 167.7 eV, and the surfaces of the HUDM-SB coatings had two S elemental peaks at 169.1 and 167.8 eV, corresponding to the S2p_1/2_ and S2p_3/2_ signal absorption peaks of the –SO_3_^−^ groups [36], respectively. Therefore, the results confirmed that the reaction of the IPUDM and HUDM precursors with 1,3-propane sulfone and tertiary amines through an opening reaction covalently linked sulfobetaine glycine to the urushiol molecules. Additionally, this synthesis resulted in zwitterionic urushiol-based polymeric antifouling coatings, rich in cationic (+NR_4_) and anionic (–SO_3_^−^) functional groups.

### 2.3. Surface and Physicochemical Properties of the Coatings

The physical and mechanical properties of zwitterionic urushiol-polymer coatings, which were synthesized by coupling urushiol molecules with different types of isocyanates, are presented in Table 1. The urushiol coating coupled with cycloaliphatic isocyanates (IPUDM-SB) exhibited superior adhesion, pencil hardness, and gloss compared to the urushiol coating coupled with aliphatic isocyanates (HUDM-SB). This observation suggests that coatings with a higher proportion of rigid structures within the urushiol polymer have enhanced hardness. Importantly, both IPUDM-SB and HUDM-SB coatings demonstrated higher adhesion and hardness than the unmodified zwitterionic urushiol-based polymer coatings, indicating that the introduction of sulfobetaine moieties did not compromise the original physical properties of the coatings.

**Figure 3:**
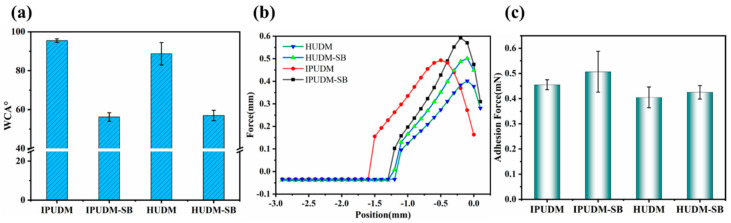
Basic performance tests for IPUDM, IPUDM−SB, HUDM and HUDM−SB coatings: (**a**) WCA, (**b**) adhesion force measurements, and (**c**) statistical adhesion force measurements. Each sample was measured thrice and averaged.

In order to evaluate the hydrophilic properties of the coated surfaces, static water contact angles (WCAs) were measured at room temperature for each of the coatings, namely, IPUDM, IPUDM-SB, HUDM, and HUDM-SB, as depicted in Figure 3. Contact-angle measurements revealed that the WCAs for IPUDM and HUDM coatings were 95.5 ± 0.9° and 88.7 ± 5.8°, respectively. The hydrophobic nature of the IPUDM coating was attributed to the presence of rigid structures originating from cycloaliphatic isocyanates. In contrast, the HUDM coating, which contained aliphatic isocyanates and saturated long-chain flexible structures, exhibited reduced hydrophobicity. The introduction of zwitterionic functionalities in the IPUDM-SB and HUDM-SB coatings led to a significant reduction in WCAs, with values of 56.2 ± 2.19° and 57.0 ± 5.77°, respectively. This finding represents respective decreases of 41.12% and 35.74% in contact angles, indicating notably enhanced hydrophilicity. This enhancement can be attributed to the abundance of anions and cations on the coating surfaces, which, through electrostatic hydration, formed a dense hydration layer without disrupting hydrogen bonding between water molecules [37]. Remarkably, IPUDM-SB coatings demonstrated the greatest increase in hydrophilicity, possibly due to the isophorone diisocyanate structure and the presence of two different NCO reactivity sites. Generally, coatings with higher hydrophilicity tend to exhibit stronger binding with water droplets. As shown in Figure 3b, the unmodified IPUDM and HUDM coatings displayed significantly lower binding with water droplets compared to the zwitterionic functionalized IPUDM-SB and HUDM-SB coatings. The adhesion forces between the IPUDM-SB and HUDM-SB coatings and water droplets measured 0.51 mN and 0.43 mN, respectively, with the IPUDM-SB coating demonstrating a higher binding force than the HUDM-SB coating, consistent with the WCA test results.

**Figure 4:**
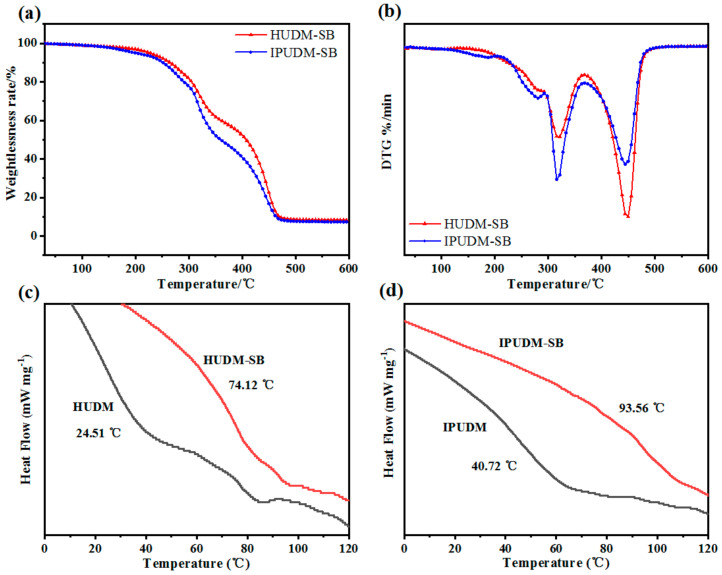
Thermal stability tests for HUDM−SB and IPUDM−SB coatings: (**a**) DTG (**b**) TGA. Glass transition temperature tests for coatings: (**c**) HUDM and HUDM−SB (**d**) IPUDM and IPUDM−SB.

The thermal stability of room-temperature-cured IPUDM-SB and HUDM-SB coatings was examined using thermogravimetric analysis (TGA), as depicted in Figure 4a,b. The results indicated that IPUDM-SB and HUDM-SB coatings began to decompose at temperatures of 199.17 °C and 222.50 °C, with residual carbon contents of 7.47% and 8.26%, respectively. The thermal decomposition can be described as follows. In the initial step, the carbonyl bonds of the carbamate groups on the polymer’s main chain undergo thermal cleavage, resulting in isocyanates, polyols, or polyphenols. In the subsequent step, the hard segments of isocyanates break down into diisocyanates. The final step involves polyol or polyphenol decomposition. This finding indicates that the breakdown of the urea bonds in the isocyanates and the carbamate groups in the hard segments occurs earlier than the decomposition of the soft segments. IPUDM-SB, which contains cycloaliphatic isocyanates, begins to decompose at a slightly lower temperature than HUDM-SB, which includes aliphatic isocyanates, indicating that IPUDM-SB has lower thermal stability.

The thermal performance of the room-temperature-cured coatings, namely IPUDM, IPUDM-SB, HUDM, and HUDM-SB, was further investigated through differential scanning calorimetry (DSC) analysis. As illustrated in Figure 4c,d, distinct glass transition temperatures (T_g_) were observed during the second heating scan in the DSC test: 40.72 °C, 93.56 °C, 24.51 °C, and 74.12 °C, respectively. It is evident that HUDM and HUDM-SB coatings have slightly lower T_g_ values compared to IPUDM and IPUDM-SB coatings. This difference can be attributed to the use of 1,6-hexamethylene diisocyanate as the crosslinker in HUDM and HUDM-SB coatings, which introduces more flexible structures and thus results in lower T_g_ values. Additionally, the DSC results reveal that IPUDM-SB and HUDM-SB coatings, which are functionalized with sulfobetaine, exhibit significantly higher T_g_ values compared to IPUDM and HUDM coatings. This significant change in T_g_ values suggests that the introduction of sulfobetaine moieties may increase cross-linking density and reduce the free volume fraction of the main chains of IPUDM-SB and HUDM-SB coatings. Furthermore, IPUDM-SB, which contains cycloaliphatic isocyanates, exhibits a slightly higher cross-linking density compared to HUDM-SB, which contains aliphatic isocyanates.

### 2.4. Surface and Physicochemical Properties of the Coatings

**Figure 5:**
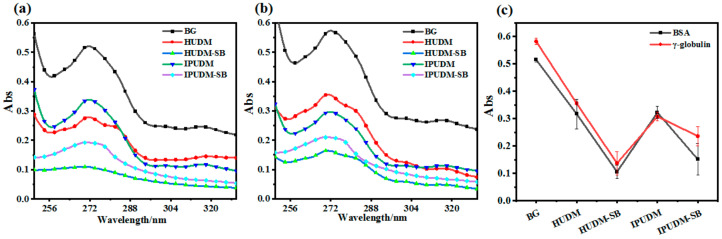
The absorbance at 280 nm of protein solutions attached to the surfaces of BG, HUDM, HUDM-SB, IPUDM, and IPUDM-SB coatings. (**a**) BSA; (**b**) γ-globulin; (**c**) Adsorption capacity of BSA and γ-globulin solutions on BG, HUDM, HUDM-SB, IPUDM, and IPUDM-SB coatings at 280 nm.

The occurrence of biofouling on the surfaces of underwater equipment involves several stages, including formation of an organic monolayer, development of a biofilm, adhesion of algal spores and protozoa, and attachment of macrofouling organisms. A crucial initial step in macrofouling is the successful adsorption of organic compounds, such as proteins, polysaccharides, and nucleic acids, onto underwater substrates. Hydrophilic antifouling coatings operate by capitalizing on the inhibitory effect of the surface hydration layer on the adsorption of organic macromolecules, which discourages subsequent attachment by fouling organisms [38]. Consequently, evaluating the anti-protein-adsorption capacity of antifouling coatings is essential for assessing their fouling resistance. In this study, we employed two major plasma proteins, bovine serum albumin (BSA) and γ-globulin, to evaluate the anti-protein-adsorption performance of the coatings through static protein adsorption. The absorbance of BSA and γ-globulin solutions to different coating surfaces is presented in Figure 5a,b. Notably, for both protein solutions, the absorbance to the IPUDM-SB and HUDM-SB coatings was lower than the absorbance to the BG, IPUDM, and HUDM coatings. As depicted in Figure 5c, for the BSA solution, the ultraviolet absorbances at 280 nm were 0.51, 0.31, and 0.32 for the BG, HUDM, and IPUDM coatings, respectively. In the case of the γ-globulin solution, the corresponding ultraviolet absorbances for these coatings were 0.58, 0.36, and 0.3. Both IPUDM and HUDM coatings exhibited significantly lower ultraviolet absorbance at 280 nm for both types of proteins compared to the blank control BG. This result indicates that IPUDM and HUDM coatings have some level of resistance to protein adhesion. After the addition of sulfobetaine zwitterionic groups, the IPUDM-SB and HUDM-SB coatings showed a significant reduction in ultraviolet absorbance at 280 nm in both BSA and γ-globulin solutions. The ultraviolet absorbance for BSA solutions decreased to 0.11 and 0.35, respectively, for the two coatings, while the ultraviolet absorbance for γ-globulin solutions decreased to 0.14 and 0.24. These findings indicate that both coatings exhibit excellent fouling resistance, primarily due to the highly hydrophilic nature of the zwitterionic groups on the coating surfaces. These groups, through electrostatically induced hydration interactions, form a hydration layer, hindering the interaction between proteins and the surface. The zwitterionic groups thus provide the coatings with outstanding anti-protein-adhesion properties [39].

### 2.5. Antibacterial Performance of the Coatings

**Figure 6:**
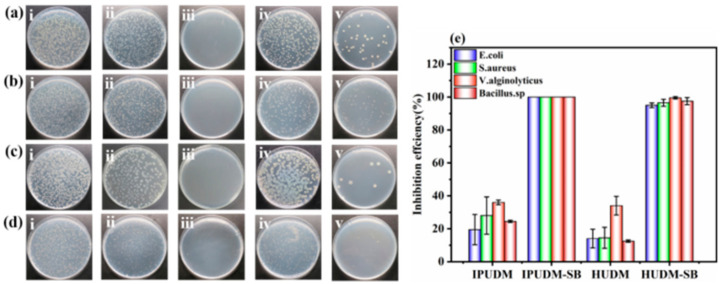
Digital photographs of antibacterial tests conducted with representative bacteria: (**a**) Gram-negative *E. coli*; (**b**) Gram-positive *S. aureus*; (**c**) Gram-negative *V. alginolyticus*, and (**d**) Gram-positive *Bacillus* sp. after 24 h of incubation on various coatings, namely (i) BG, (ii) IPUDM, (iii) IPUDM-SB, (iv) HUDM, and (v) HUDM-SB. Additionally, (**e**) the antibacterial efficiency of IPUDM, IPUDM-SB, HUDM, and HUDM-SB coatings was compared to BG in terms of their effects on *E. coli*, *S. aureus*, *V. alginolyticus*, and *Bacillus* sp., based on three parallel experiments.

To investigate the antibacterial performance of IPUDM-SB and HUDM-SB coatings, four bacterial strains were employed for assessment, namely, Gram-negative *E. coli* (BW 25113), Gram-positive *S. aureus* (ATCC 25923), marine bacterium *V. alginolyticus* (ATCC 33787), and *Bacillus* sp. (MCCC 1B00342). The antibacterial effectiveness of the antifouling coatings was assessed by determining the number of bacterial colonies that developed on agar plates from a bacterial culture broth collected from the sample surface and comparing to the yield from the control sample BG. As shown in Figure 6a, visual images of agar plates from various samples clearly depict substantial numbers of bacterial colonies grown from culture broth collected from the unmodified IPUDM and HUDM coatings, indicating limited antibacterial properties. The antibacterial rates of IPUDM coatings against *E. coli*, *S. aureus*, *V. alginolyticus*, and *Bacillus* sp. were 19.5%, 28%, 36%, and 24.5%, respectively, while HUDM coatings displayed lower antibacterial effectiveness rates of 14%, 14.5%, 34%, and 12.5% against the same bacteria (Figure 6b). In contrast, IPUDM-SB coatings exhibited the highest antibacterial effectiveness against all four bacteria, with nearly no bacterial colonies on the agar plates and an antibacterial effectiveness rate of 99.9%. On the other hand, growth media collected from the HUDM-SB coatings yielded scattered bacterial colonies on agar plates for all four bacteria, with antibacterial effectiveness rates of 95.0%, 96.5%, 99.5%, and 97.5% (Figure 6b). The zwitterionic sulfobetaine-modified polyphenol coatings thus demonstrated remarkable antibacterial properties. This finding can be mainly attributed to the cationic quaternary ammonium salts on the surfaces of IPUDM-SB and HUDM-SB coatings, which actively engage negatively charged bacteria through electrostatic interactions, resulting in cell-membrane rupture, cytoplasm leakage, and bacterial death. The ions thus exert antibacterial effects [40,41].

### 2.6. Algal-Fouling Resistance of the Coatings

**Figure 7:**
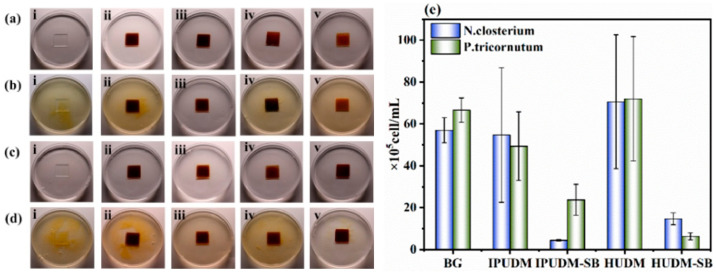
Photographs depicting algal inhibition: 1 day—(**a**) *N. closterium* and (**c**) *P. tricornutum*; 7 days—(**b**) *N. closterium* and (**d**) *P. tricornutum* in an f/2 medium with coatings (i) BG, (ii) IPUDM, (iii) IPUDM-SB, (iv) HUDM, and (v) HUDM-SB. Additionally, (**e**) shows the concentrations of *N. closterium* and *P. tricornutum* determined through cell counting with a hemocytometer after 7 days of cultivation, based on three parallel experiments.

Marine environments harbor abundant marine microalgae that readily adhere to marine structure surfaces, forming conducive biofilms that provide favorable conditions for the subsequent attachment of larger marine fouling organisms. Consequently, in this study, two marine microalgae, *N. closterium* and *P. tricornutum*, served as models for marine fouling. We investigated the anti-biofouling capacity of zwitterionic-functionalized polyphenol coatings. As illustrated in Figure 7, BG, IPUDM, IPUDM-SB, HUDM, and HUDM-SB coatings were immersed in diluted marine microalgae cell solutions in an f/2 culture medium for co-culture. As shown in the images in Figure 7a–d, after 7 days of immersion, a substantial amount of greenish-yellow precipitate was observed at the bottom of the culture medium with the BG, IPUDM, and HUDM coatings. This result suggests extensive growth and proliferation of *N. closterium* and *P. tricornutum* in the culture media with these three samples. Furthermore, the concentrations of marine microalgae cells in the culture media with the IPUDM and HUDM coatings did not differ significantly from that in culture with the blank control BG, indicating that these two coatings did not inhibit algal cell proliferation, a finding that agrees with the results of the antibacterial assay. Before sulfobetaine was introduced, the IPUDM and HUDM coatings lacked effective antibacterial and anti-algal functional groups, and as a result, they did not exhibit resistance to fouling. However, after sulfobetaine was incorporated into the IPUDM and HUDM coatings, the IPUDM-SB and HUDM-SB coatings significantly inhibited the growth of both marine microalgae species. As shown in Figure 7e, the concentration of *N. closterium* cells in the IPUDM-SB coating culture medium was 4.45 × 10^5^ cells/mL and the concentration of *P. tricornutum* cells was 23.75 × 10^5^ cells/mL. In the culture media with HUDM-SB coatings, the concentration of *N. closterium* cells was 14.7 × 10^5^ cells/mL, and the concentration of *P. tricornutum* cells was 6.31 × 10^5^ cells/mL. Furthermore, after 7 days of immersion in water, IPUDM-SB and HUDM-SB coatings remained intact and did not exhibit significant signs of swelling, indicating their good stability underwater.

**Figure 8:**
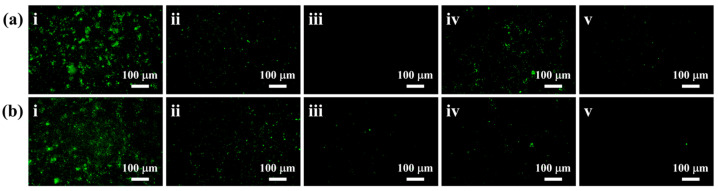

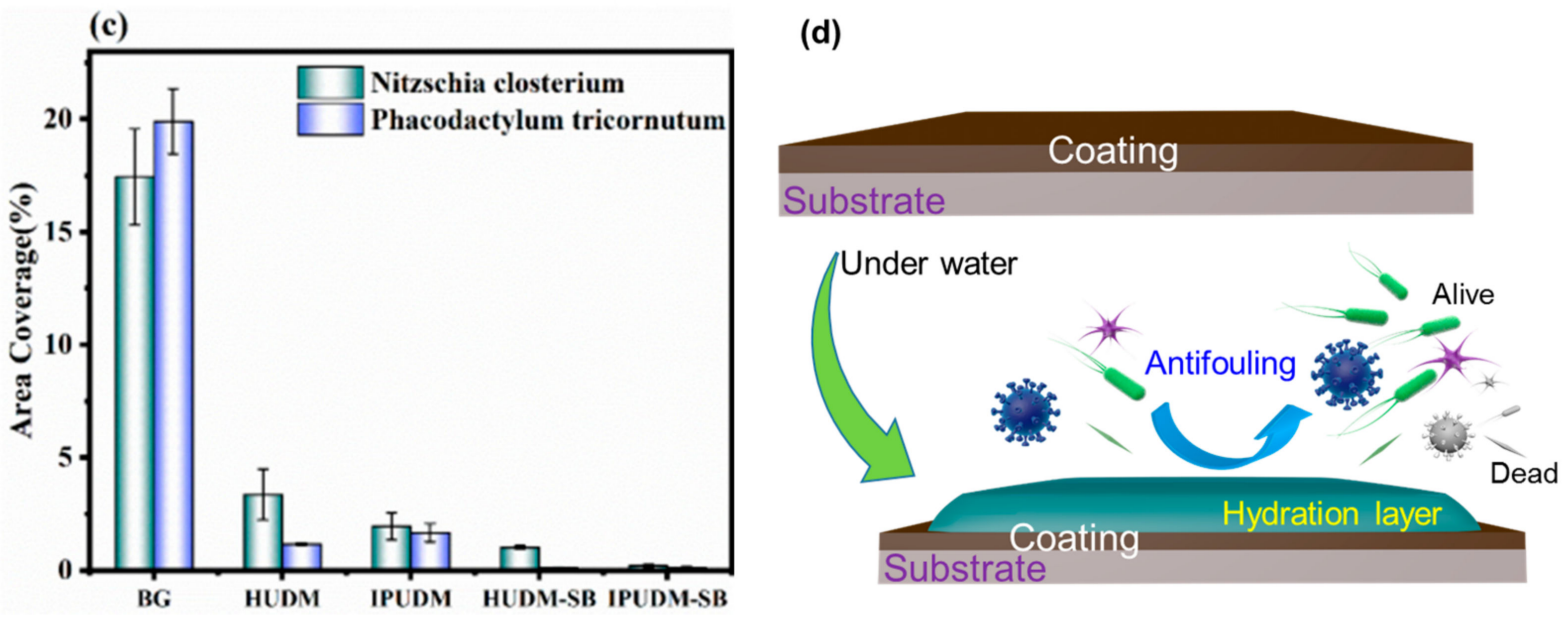
Fluorescent photographs of (**a**) *Nannochloropsis closterium* and (**b**) *Phaeodactylum tricornutum* adhesion after 7 days of cultivation on coatings (i) BG, (ii) IPUDM, (iii) IPUDM-SB, (iv) HUDM, and (v) HUDM-SB. The scale bars in the images represent 40 µm. (**c**) Statistical chart of algal density in examined fields using ImageJ software, based on five random fields at 40× magnification, covering 0.156 mm^2^ per field. (**d**) Antifouling mechanism of the HUDM-SB and IPUDM-SB coatings.

For a more comprehensive analysis of the adhesion of marine algal cells on zwitterionic-functionalized polyphenol coatings, samples coated with these materials were immersed in diluted algal cell solutions for 7 days, then examined using fluorescence microscopy. Figure 8a,b depicts fluorescence microscopy images of *N. closterium* and *P. tricornutum* adhered to the BG, IPUDM, IPUDM-SB, HUDM, and HUDM-SB coatings. To quantify the coverage of algal cells on the coating surface, we measured the fluorescence intensity of the images using ImageJ (1.52a) software. The fluorescence microscopy images of the BG surface showed a consistent and bright fluorescence, indicating the uniform adhesion of a large number of algal cells to the surface, with coverages of 17.14% for *N. closterium* and 19.88% for *P. tricornutum* (Figure 8c). On the surfaces of the IPUDM and HUDM coatings, the coverage values of *N. closterium* were 1.95% and 3.36%, respectively, and those of *P. tricornutum* were 1.67% and 1.17%, respectively (Figure 8c). In contrast, the coverage values of *N. closterium* on the surfaces of the IPUDM-SB and HUDM-SB coatings were only 0.21% and 1.02%, while those of *P. tricornutum* were only 0.13% and 0.10% (Figure 8c). These results indicate that fewer algal cells adhered to the surfaces of the IPUDM-SB and HUDM-SB coatings, highlighting their superior anti-algal-adhesion properties. These findings can be attributed to several factors. First, the sulfobetaine groups on the surfaces of the IPUDM-SB and HUDM-SB coatings effectively inhibit the growth and proliferation of algal cells, reducing algal cell concentrations [42,43]. Second, as depicted in Figure 8d, the hydrophilicity of the IPUDM-SB and HUDM-SB coatings, combined with the dense hydration layer formed on their surfaces through electrostatic interactions, reduces the strength of adhesive forces between marine algal cells and the coatings [44]. It is thus easier to rinse the cells off with deionized water, resulting in improved resistance to algal cell adhesion.

## 3. Materials and Methods

### 3.1. Materials

Raw lacquer, purchased from Maoba Town, Hubei Province, China, was treated with ethanol to extract urushiol [45]. Hexamethylene diisocyanate (HMDI), isophorone diisocyanate (IPDI), dibutyltin dilaurate (DBTDL), trichloromethane, and N,N-dimethylethanolamine (DMEA) were purchased from Shanghai McLin Biochemical Co., Ltd. (Shanghai, China). Tetrahydrofuran, ethanol, 1,3-propanesultone, deionized water, BSA, and γ-globulin were obtained from Shanghai National Pharmaceutical Reagent Co., Ltd. (Shanghai, China). Phosphate-buffered saline (PBS, pH = 7.4) was procured from Shanghai Sangon Biotech Co., Ltd. (Shanghai, China). Artificial seawater (ASW) was prepared according to ASTM D1141–1998 (2013). *E. coli*, *V. alginolyticus*, *S. aureus*, and *Bacillus*. sp. were purchased from Beijing Baibo Wei Biotechnology Co., Ltd. (Beijing, China). *N. closterium* and *P. tricornutum* were obtained from Shanghai Guangyu Biotechnology Co., Ltd. (Shanghai, China).

### 3.2. Synthesis of HUDM-SB Monomers

Under a nitrogen atmosphere, 1.78 g of DMEA (0.20 mol), 3.36 g of HMDI (0.20 mol), and 20 mL of anhydrous trichloromethane were placed in a 250 mL three-necked round-bottom flask equipped with a thermometer, a reflux condenser, and a constant-pressure dropping funnel. Then, the mixture was stirred continuously at 40 °C for 2 h to facilitate the addition reaction between the alcohol hydroxyl and the NCO groups of DMEA and HMDI, respectively, forming a linear precursor (PHDE). Subsequently, 6.32 g of urushiol (0.20 mol) dissolved in 10 mL of anhydrous trichloromethane was slowly added to the aforementioned reaction system. The reaction system was then heated to 70 °C, and the reaction was allowed to proceed for an additional 12 h. The system was cooled to room temperature after the reaction was completed, and rotary evaporation was employed to remove the solvent and obtain the urushiol-based precursor (HUDM). Next, HUDM and 1,3-propanesultone were mixed in a 1:1 molar ratio in a 100 mL three-necked flask. The mixture was stirred continuously at 37 °C for 24 h, and rotary evaporation was used to remove the solvent. Finally, a black viscous liquid, the urushiol-based sulfobetaine monomer (HUDM-SB), was obtained by vacuum drying.

### 3.3. Synthesis of IPUDM-SB Monomers

In a nitrogen atmosphere, 1.78 g of DMEA (0.20 mol), 3.36 g of IPDI (0.20 mol), and 20 mL of anhydrous trichloromethane were placed in a 250 mL three-necked round-bottom flask equipped with a thermometer, a reflux condenser, and a constant-pressure dropping funnel. The mixture was stirred continuously at 40 °C for 2 h, facilitating the addition reaction between the alcohol hydroxyl and the NCO groups of DMEA and IPDI, respectively, resulting in the formation of a linear precursor (PIPDE). Subsequently, 6.32 g of urushiol (0.20 mol) dissolved in 10 mL of anhydrous trichloromethane was added slowly to the aforementioned reaction system. The reaction system was then heated to 70 °C, and the reaction was allowed to proceed for an additional 12 h. When the reaction was complete, the system was cooled to room temperature and rotary evaporation was employed to remove the solvent and obtain the urushiol-based precursor (IPUDM). Next, IPUDM and 1,3-propanesultone were mixed in a 1:1 molar ratio in a 100 mL three-necked flask. The mixture was stirred continuously at 37 °C for 24 h, and rotary evaporation was used to remove the solvent. Finally, a black viscous liquid, the urushiol-based sulfobetaine monomer (IPUDM-SB), was obtained after vacuum drying.

### 3.4. Synthesis of HUDM-SB and IPUDM-SB Coatings

First, 2.5 cm × 2.5 cm slides of bare glass (BG) underwent sequential cleaning through ultrasonication in acetone, ethanol, and deionized water for 10 min, which was followed by N_2_ drying. A predetermined quantity of HUDM-SB or IPUDM-SB was dissolved in tetrahydrofuran (40 wt%), and curing agent DBTDL (0.05 wt%) was incorporated into the solution. After thorough stirring, the solution was drop-cast onto the BG slides and left at room temperature until the solvent evaporated completely, resulting in the formation of cured coatings. HUDM and IPUDM coatings were prepared as control samples using the same method.

### 3.5. Characterizations

The chemical structures of the HUDM, IPUDM, HUDM-SB, and IPUDM-SB monomers were characterized using Fourier-transform infrared (FTIR) spectroscopy (Thermo Fisher Nicolet 5700, Waltham, MA, USA). The spectra were recorded in the 4000–400 cm^−1^ range with 4 cm^−1^ resolution and 32 scans. The composition of the surface elements and contents of the HUDM, IPUDM, HUDM-SB, and IPUDM-SB coatings were characterized using X-ray photoelectron spectroscopy (XPS, Thermo Electron Fisher VG MultiLab 2000, Waltham, MA, USA) with monochromatic Al Kα radiation (1254.0 eV). The binding energies were calibrated using the C1s peak at 284.8 eV. The hydrophilicity of the surfaces of the different coatings was assessed using static water contact angle measurements (DSA25, Kruss, Hamburg, Germany). Deionized water (2 µL) was added at room temperature to conduct the measurements, with five measurements taken for each sample to obtain average values and standard deviations. The glass transition temperatures of the coatings were determined using differential scanning calorimetry (DSC, Mettler-Toledo 3, Greifensee, Switzerland). The measurements were conducted under a nitrogen atmosphere with a 50 mL/min flow rate and a 10 °C/min heating rate from −30 to 160 °C. The thermal stability of the coatings was analyzed by thermogravimetric analysis (TGA, Mettler-Toledo 3, Greifensee, Switzerland). The measurements were conducted under a nitrogen atmosphere with a 50 mL/min flow rate and a 10 °C/min heating rate from 30 to 600 °C. The coating adhesion of droplets was measured using a surface-tension analyzer (Kruss K100MK2, Hamburg, Germany). Deionized water (6 µL) was used at room temperature to conduct the measurements with an immersion depth and time of 0.1 mm and 0.01 min, respectively, and five measurements were conducted for each sample to obtain average values and standard deviations. The pencil-scratch method was used to test the hardness of the coatings according to the GB/T 6739–2006/ ISO 15184–2012 standards [46]. The cross-cut method was used to assess the adhesion of the coatings according to the GB/T 1720–1979 standard [47].

### 3.6. Testing of Surface-Protein Adsorption 

Protein-adhesion experiments were conducted according to the established protocols described in the references. The Micro BCATM protein assay was utilized to measure the adhesion of samples to BSA. Initially, the coating samples, including BG, HUDM, IPUDM, HUDM-SB, and IPUDM-SB (2.5 cm × 2.5 cm), were sterilized under ultraviolet (UV) light for 30 min. Subsequently, the samples were equilibrated in sterilized PBS for 2 h. Next, the samples were immersed in a protein solution with a 2 mg/mL concentration. Subsequently, the samples were incubated at 37 °C for 4 h and gently rinsed three times with PBS. They were then placed in 4 mL of PBS containing 2 wt% sodium dodecyl sulfate (SDS) and subjected to 30 min of ultrasonication in the SDS solution to remove any BSA that had not adhered to the surface of the coatings. The absorbance at 280 nm was measured using ultraviolet–visible (UV–vis) spectroscopy (TU-1810, Beijing Purkinje General Instrument Co., Beijing, China) to determine the concentration of BSA that had adhered to the surface of the coatings. The resistance of each coating to protein adsorption by γ-globulin was evaluated by the same methodology.

### 3.7. Assessment of Antibacterial Activity 

The antibacterial activity of the coatings was evaluated against four bacterial strains: Gram-negative *E. coli* (BW 25113) and *V. alginolyticus* (ATCC 33787), and Gram-positive *S. aureus* (ATCC 25923) and *Bacillus*. sp. (MCCC 1B00342). Strains of *E. coli* and *S. aureus* were preserved in a 1:1 solution of Luria–Bertani (LB) broth and 40% (*v*/*v*) glycerol and stored at −80 °C. Strains of marine bacterium *V. alginolyticus* and *Bacillus*. sp. were preserved in a 1:1 solution of 2216E medium and 40% (*v*/*v*) glycerol and stored at −80 °C. The bacterial strains were cultivated in fresh LB broth (2216E medium for marine bacterium) at 37 °C (30 and 28 °C for *V. alginolyticus* and *Bacillus*. sp., respectively) before the antibacterial experiments, with shaking at 170 rpm for 20 h until O.D._600nm_ reached 1.8–2.0. Subsequently, BG slides and HUDM, IPUDM, HUDM-SB, and IPUDM-SB plates (2.5 cm × 2.5 cm) were wiped with anhydrous ethanol, disinfected using 30 min of UV irradiation, and placed in plastic Petri dishes. The bacteria were diluted to approximately 10^5^–10^6^ CFU/mL with fresh LB broth (2216E medium for marine bacterium) to prepare the bacterial suspensions, and 100 µL of the diluted bacterial suspension was inoculated onto the BG slides and onto the HUDM, IPUDM, HUDM-SB, and IPUDM-SB coatings. The samples were covered with plastic wrap and incubated at 37 °C for 24 h. Subsequently, the samples were gently rinsed with 10 mL of sterile PBS to remove the non-adherent bacteria. The antibacterial rate (*A.R.*) of the HUDM, IPUDM, HUDM-SB, and IPUDM-SB coatings was calculated based on the count of bacterial colonies in the PBS rinse solution on agar plates using the following formula:

$$A.R.=\frac{N_{contriol}-N_{sample}}{N_{control}}\times 100\%$$

where *N_control_* represents the colony count (in CFU/mL) on BG plates and *N_sample_* represents the colony count (in CFU/mL) on the HUDM, IPUDM, HUDM-SB, and IPUDM-SB coatings. Each sample was measured three times to obtain the average and the standard deviation.

### 3.8. Algal Biofouling Assessment

Algal growth and attachment experiments were conducted to evaluate the effects of HUDM-SB and IPUDM-SB coatings on algae. Algal cells, specifically *N. closterium* and *P. tricornutum*, were cultured in an f/2 medium using ASW with a 22 ± 2 °C growth temperature and a 12 h:12 h light:dark cycle. The algal cells were diluted to a concentration of 10^5^–10^6^ cells/mL with fresh culture medium for use in algal cell reproduction and adhesion experiments after seven days of cultivation. BG slides and HUDM, IPUDM, HUDM-SB, and IPUDM-SB coatings were sterilized under UV light for 30 min and immersed in 30 mL of culture medium containing algal cells. The concentration of algal cells was determined using a hemocytometer after seven days of immersion, and the images were recorded to monitor algal growth. The BG slides and the HUDM, IPUDM, HUDM-SB, and IPUDM-SB coatings were removed from the experimental culture medium following the cultivation period and rinsed with 20 mL of sterile PBS solution to remove all non-adherent algal cells. Subsequently, the algae adhered onto the surfaces of BG slides, HUDM, IPUDM, HUDM-SB, and IPUDM-SB coatings were examined using an Epi-illumination fluorescence microscope (Eclipse Ci-L plus, Nikon, Tokyo, Japan) with a DAPI fluorescence filter kit. The images of five random fields (20× magnification, 0.156 mm^2^/per field) under a dark field were recorded for each sample. ImageJ software was employed to analyze the fluorescence microscopy images and determine the algal coverage on the surfaces of the BG slides and the HUDM, IPUDM, HUDM-SB, and IPUDM-SB coatings. Three parallel experiments were conducted to calculate the standard deviation.

## 4. Conclusions

In summary, environmentally friendly sulfobetaine-functionalized polyurushiol coatings, namely HUDM-SB and IPUDM-SB, were successfully synthesized. These coatings utilize sustainable natural urushiol as the primary material and two types of diisocyanates as crosslinkers. They incorporate zwitterionic sulfobetaine, which has remarkable water affinity, as their hydrophilic component. These zwitterions form a dense hydration layer underwater, effectively deterring the adhesion of nonspecific proteins, marine microalgae, and other fouling organisms. The introduction of sulfobetaine significantly enhances the hydrophilicity of polyurushiol coatings, resulting in water contact angles of 56.2° and 57.0° for the HUDM-SB and IPUDM-SB coatings, respectively. These results demonstrate the coatings’ excellent resistance to protein adhesion. Furthermore, the sulfobetaine moieties on the coating surfaces actively engage with negatively charged bacteria through electrostatic interactions, rupturing their cell membranes and resulting in superior antibacterial properties. The IPUDM-SB coatings demonstrate remarkable antibacterial activity, with 99.9% prevention of the growth of prevalent Gram-negative bacteria (*E. coli* and *V. alginolyticus*) and Gram-positive bacteria (*S. aureus* and *Bacillus* sp.). Similarly, the HUDM-SB coatings also exhibit significant antibacterial activity, achieving rates exceeding 95.0% against these four bacterial species. Moreover, the sulfobetaine moieties on the coating surfaces effectively inhibit the growth and proliferation of algal cells. Additionally, they create a dense hydration layer through electrostatic interactions, reducing adhesive forces between marine microalgae and the coating surfaces. This layer effectively prevents biofouling by marine microalgae without the use of fouling-release agents. These coatings offer a green and environmentally friendly antifouling strategy, addressing economic losses caused by marine biofouling and environmental pollution arising from the misuse of fouling-release agents.

## Figures and Tables

**Table 1:** Physical and mechanical properties of different coatings.

Samples	Adhesion (Grade)	Pencil Hardness	Glossiness (%)
IPUDM	7	1H	93.7
IPUDM-SB	3	2H	88.7
HUDM	6	4B	65.8
HUDM-SB	4	HB	44.6

## Data Availability

The data presented in this study are available on request from the corresponding author.

## References

[1] Jin H., Tian L., Zhao J., Ren L. (2022). Bioinspired marine antifouling coatings: Status, prospects, and future. Prog. Mater. Sci..

[2] Pourhashem S., Seif A., Saba F., Nezhad E.G., Ji X., Zhou Z., Zhai X., Mirzaee M., Duan J., Rashidi A. (2022). Antifouling nanocomposite polymer coatings for marine applications: A review on experiments, mechanisms, and theoretical studies. J. Mater. Sci. Technol..

[3] Romeu M.J., Mergulhão F. (2023). Development of Antifouling Strategies for Marine Applications. Microorganisms.

[4] Ma C., Wang W., Li W., Sun T., Feng H., Lv G., Chen S. (2023). Full solar spectrum-driven Cu_2_O/PDINH heterostructure with enhanced photocatalytic antibacterial activity and mechanism insight. J. Hazard. Mater..

[5] Ding T., Xu L. (2022). A Cu_2_O-based marine antifouling coating with controlled release of copper ion mediated by amphiphilic PLMA-b-PDMAEMA copolymers. Prog. Org. Coat..

[6] Soon Z.Y., Jung J., Jang M., Kang J., Jang M., Lee J., Kim M. (2019). Zinc pyrithione (ZnPT) as an antifouling biocide in the marine environment—A literature review of its toxicity, environmental fates, and analytical methods. Water Air Soil Pollut..

[7] Lee S., Haque M.N., Lee D., Rhee J. (2023). Comparison of the effects of sublethal concentrations of biofoulants, copper pyrithione and zinc pyrithione on a marine mysid-A multigenerational study. Comp. Biochem. Physiol. Part C Toxicol. Pharmacol..

[8] Almond K.M., Trombetta L.D. (2016). The effects of copper pyrithione, an antifouling agent, on developing zebrafish embryos. Ecotoxicology.

[9] Li Q., Huang M., Li F., Ling Z., Meng Y., Chen F., Ji Z., Wang S. (2024). Biomimetic stable cellulose based superhydrophobic Janus paper sheets engineered with industrial lignin residues/nano-silica for efficient oil-water separation. Ind. Crops Prod..

[10] Jiang X., Li Q., Li X., Meng Y., Ling Z., Ji Z., Chen F. (2022). Preparation and Characterization of Degradable Cellulose−Based Paper with Superhydrophobic, Antibacterial, and Barrier Properties for Food Packaging. Int. J. Mol. Sci..

[11] Chen J., Jian R., Yang K., Bai W., Huang C., Lin Y., Zheng B., Wei F., Lin Q., Xu Y. (2021). Urushiol-based benzoxazine copper polymer with low surface energy, strong substrate adhesion and antibacterial for marine antifouling application. J. Clean. Prod..

[12] Sathishkumar G., Gopinath K., Zhang K., Kang E.T., Xu L., Yu Y. (2022). Recent progress in tannic acid-driven antibacterial/antifouling surface coating strategies. J. Mater. Chem. B.

[13] Chen J., Zhao J., Lin F., Zheng X., Jian R., Lin Y., Wei F., Lin Q., Bai W., Xu Y. (2023). Polymerized tung oil toughened urushiol-based benzoxazine copper polymer coatings with excellent antifouling performances. Prog. Org. Coat..

[14] Zhang C., Qi Y., Zhang S., Xiong G., Wang K., Zhang Z. (2023). Anti-marine biofouling adhesion performance and mechanism of PDMS fouling-release coating containing PS-PEG hydrogel. Mar. Pollut. Bull..

[15] Wang F., Zhang H., Yu B., Wang S., Shen Y., Cong H. (2020). Review of the research on anti-protein fouling coatings materials. Prog. Org. Coat..

[16] Li K., Qi Y., Zhou Y., Sun X., Zhang Z. (2021). Microstructure and properties of poly (ethylene glycol)-segmented polyurethane antifouling coatings after immersion in seawater. Polymers.

[17] Sun D., Li P., Li X., Wang X. (2020). Protein-resistant surface based on zwitterion-functionalized nanoparticles for marine antifouling applications. New J. Chem..

[18] Ye Z., Zhang P., Zhang J., Deng L., Zhang J., Lin C., Guo R., Dong A. (2019). Novel dual-functional coating with underwater self-healing and anti-protein-fouling properties by combining two kinds of microcapsules and a zwitterionic copolymer. Prog. Org. Coat..

[19] Zheng L., Sundaram H.S., Wei Z., Li C., Yuan Z. (2017). Applications of zwitterionic polymers. React. Funct. Polym..

[20] Chen P., Lang J., Zhou Y., Khlyustova A., Zhang Z., Ma X., Liu S., Cheng Y., Yang R. (2022). An imidazolium-based zwitterionic polymer for antiviral and antibacterial dual functional coatings. Sci. Adv..

[21] Zhang J., Wu M., Peng P., Liu J., Lu J., Qian S., Feng J. (2022). “Self-Defensive” antifouling zwitterionic hydrogel coatings on polymeric substrates. ACS Appl. Mater. Interfaces.

[22] Zhang H., Li Y., Tian S., Qi X., Yang J., Li Q., Lin C., Zhang J., Zhang L. (2022). A switchable zwitterionic ester and capsaicin copolymer for multifunctional marine antibiofouling coating. Chem. Eng. J..

[23] Guan Y., Li S.-L., Fu Z., Qin Y., Wang J., Gong G., Hu Y. (2022). Preparation of antifouling TFC RO membranes by facile grafting zwitterionic polymer PEI-CA. Desalination.

[24] Li D., Li K., Fang J. (2022). Research Progress on Modification and Application of Raw Lacquer. ChemistrySelect.

[25] Chen Y., Zhang G., Zhang G., Ma C. (2021). Rapid curing and self-stratifying lacquer coating with antifouling and anticorrosive properties. Chem. Eng. J..

[26] Chen S., Wang L., Lin X., Ni P., Liu H., Li S. (2023). Catechol derivative urushiol’s reactivity and applications beyond traditional coating. Ind. Crops Prod..

[27] Ma J., Lee G.H., Kim J.H., Kim S.W., Jo S., Kim C. (2022). A transparent self-healing polyurethane–isophorone-diisocyanate elastomer based on hydrogen-bonding interactions. ACS Appl. Polym. Mater..

[28] Oh J., Kim Y.K., Hwang S., Kim H., Jung J., Jeon C., Kim J., Lim S.K. (2022). Synthesis of thermoplastic polyurethanes containing bio-based polyester polyol and their fiber property. Polymers.

[29] Gaddam S.K., Arukula R. (2022). Renewable soft segment-induced anionic waterborne polyurethane dispersions with enriched bio-content. J. Polym. Res..

[30] Cheng Q., Jia X., Cheng P., Zhou P., Hu W., Cheng C., Hu H., Xia M., Liu K., Wang D. (2022). Improvement of the filtration and antifouling performance of a nanofibrous sterile membrane by a one-step grafting zwitterionic compound. New J. Chem..

[31] Yuan Y., Tan W., Zhang J., Li Q., Guo Z. (2022). Water-soluble amino functionalized chitosan: Preparation, characterization, antioxidant and antibacterial activities. Int. J. Biol. Macromol..

[32] Zhao J., Chen J., Zheng X., Lin Q., Zheng G., Xu Y., Lin F. (2023). Urushiol-Based Benzoxazine Containing Sulfobetaine Groups for Sustainable Marine Antifouling Applications. Polymers.

[33] Morang S., Karak N. (2022). Nanocomposites of waterborne polyurethanes. Eco-Friendly Waterborne Polyurethanes.

[34] Bi J., Yan Z., Hao L., Elnaggar A.Y., El-Bahy S.M., Zhang F., Azab I.H.E., Shao Q., Mersal G.A., Wang J. (2023). Improving water resistance and mechanical properties of waterborne acrylic resin modified by octafluoropentyl methacrylate. J. Mater. Sci..

[35] Chou Y.-N., Ou M. (2023). Zwitterionic Surface Modification of Aldehydated Sulfobetaine Copolymers for the Formation of Bioinert Interfaces. ACS Appl. Polym. Mater..

[36] Liu Z., Xiao Y., Ma X., Geng X., Ye L., Zhang A., Feng Z. (2022). Preparation and characterisation of zwitterionic sulfobetaine containing siloxane-based biostable polyurethanes. Mater. Adv..

[37] Chen Z. (2022). Surface hydration and antifouling activity of zwitterionic polymers. Langmuir.

[38] Wang T., Zhang J., Cai Y., Xu L., Yi L. (2022). Protein-resistant amphiphilic copolymers containing fluorosiloxane side chains with controllable length. ACS Appl. Polym. Mater..

[39] Sarker P., Lu T., Liu D., Wu G., Chen H., Sajib M.S.J., Jiang S., Chen Z., Wei T. (2023). Hydration Behaviors of Nonfouling Zwitterionic Materials. Chem. Sci..

[40] Chen C., Li Z., Li X., Kuang C., Liu X., Song Z., Liu H., Shan Y. (2022). Dual-functional antimicrobial coating based on the combination of zwitterionic and quaternary ammonium cation from rosin acid. Compos. Part B.

[41] Peng J., Li K., Du Y., Yi F., Wu L., Liu G. (2023). A robust mixed-charge zwitterionic polyurethane coating integrated with antibacterial and anticoagulant functions for interventional blood-contacting devices. J. Mater. Chem. B.

[42] Malouch D., Berchel M., Dreanno C., Stachowski-Haberkorn S., Chalopin M., Godfrin Y., Jaffrès P. (2023). Evaluation of lipophosphoramidates-based amphiphilic compounds on the formation of biofilms of marine bacteria. Biofouling.

[43] Zou W., Gu J., Li J., Wang Y., Chen S. (2022). Tailorable antibacterial and cytotoxic chitosan derivatives by introducing quaternary ammonium salt and sulfobetaine. Int. J. Biol. Macromol..

[44] Qu K., Yuan Z., Wang Y., Song Z., Gong X., Zhao Y., Mu Q., Zhan Q., Xu W., Wang L. (2022). Structures, properties, and applications of zwitterionic polymers. ChemPhysMater.

[45] Xu H., Lu Z., Zhang G. (2012). Synthesis and properties of thermosetting resin based on urushiol. RSC Adv..

[46] (2012). Paints and Varnishes-Determination of Film Hardness by Pencil Test.

[47] (1979). Method of Test for Adhesion of Paint Films.

